# Book Reviews

**Published:** 1826-06

**Authors:** 


### CRITICAL ANALYSIS
### OF
### ENGLISH AND FOREIGN LITERATURE,
### RELATIVE TO THE VARIOUS BRANCHES OF
### Medical Science.

Quæ laudanda forent, et quæ culpanda, vicissim

Illa, prius, cretâ; mox hæc, carbone, *notamus*.—PERSIUS.

### DIVISION I.

### ENGLISH.

ART. I.—*Practical Observations on the Convulsions of Infants.* By JOHN NORTH, Surgeon-Accoucheur, Member of the Royal College of Surgeons.—8vo. pp. 282. Burgess and Hill, London, 1826.

THE late very distinguished Dr. JOHN CLARK, in his Commentaries on the Diseases of Children, laid it down, "that, in every case of convulsion, the brain is at the time organically affected, directly or indirectly;" and it can scarcely be unknown, at least to many of our readers, that the doctrines of this practitioner still continue to influence many of our brethren, some of them holding the highest rank in the same department. The natural consequence of this pathology is a corresponding activity in the treatment, which has principally, if not exclusively, consisted in the general or local detraction of blood, and the administration of calomel in full and frequently repeated doses. Such are the leading doctrines of Dr. Clark and his followers, and the principal object of the work before us seems to be, to enter a protest against the correctness of the theory, and, as a necessary consequence, the propriety of the practice.

Until the appearance of the volume now under consideration, we are not aware of any English author having written specifically on the convulsions of children, although several works have emanated from the French press; the most recent of which, that of M. BRACHET "sur les Causes des Convulsions chez les Enfans, et sur les Moyens d'y Remedier," now lies before us: and, before we commence our analysis of Mr. North's work, we may observe that Brachet adopts what may be termed a modification of Dr. Clark's opinion, inasmuch as he holds the convulsions of children to be always dependent upon "irritation, either primary or secondary, of the encephalon." This irritation, we are informed, may exist in different degrees, and leave behind traces which vary in distinctness, or may be sometimes entirely wanting; for this author distinctly states as his opinion, that it is not necessary for the cerebral tissue to be altered, even in cases of convulsions which have proved fatal. We shall presently find that the author more immediately before us differs in his opinions from both, although less from Brachet than from Clark, as may be gathered from what we have already said.

The first chapter of the "Practical Observations" embraces a variety of important subjects: it relates to the frequency, the causes, the symptoms, the prognosis, and the appearance on dissection, in the convulsions of infants. The discussion of these questions occupies nearly half the volume; it is full and elaborate, evincing considerable research, and containing numerous references to the opinions of the best authorities, as well as abundant proofs of original observation.

The *frequency* of convulsions, at least as a fatal complaint, does not seem to us very easily ascertained, because when they occur in the course of any disease which proves fatal, (and there are few serious diseases of infants in which they may not,) it is scarcely a legitimate inference to attribute the death of the patient to what in reality has been only one of the symptoms. It is stated by Dr. Clarke, that, of 17,650 children born at the Lying-in Hospital in Dublin, one-sixth perished during the first year, and "that nineteen out of twenty fell victims to convulsions;" while Dr. Lange has recorded that, "during thirteen years, no less than 12,769 children perished from epilepsy" at Copenhagen. These calculations, we suspect, would lead to a very exaggerated idea of the fatality of convulsions as an independent disease. It is true, however, as observed by Mr. North, that—

"Every allowance, however, being made for these probably erroneous records, it cannot be denied that a vast number of children are suddenly cut off by an unexpected attack of convulsions, which not unfrequently occurs when no previous derangement of health could be discovered, or at a time when any malady, under which the child was labouring, began to assume a less severe aspect, and to justify a confident and favourable prognosis. The occurrence of such cases is no less distressing to the parents than prejudicial to the practitioner. We never escape from censure when we have raised hopes which are suddenly and unexpectedly blasted, however impossible it might have been for human foresight to anticipate an unfavourable termination. Although, then, the convulsions of children are in most cases symptomatic of some other disease, they are of sufficient importance to admit of a separate consideration, inasmuch as they not unfrequently destroy life in a few moments, when it was not endangered by the malady which excited them. Notwithstanding, however, the universal admission of the fact, that convulsions are always to be regarded with apprehension, I am inclined to believe, from frequent personal observation, that no cases are dismissed in general practice with less attention than those which pass under the convenient term of *convulsions*." (P. 3, 4.)

It is, indeed, too much the custom with practitioners to content themselves with classing all spasmodic affections of infants under the general name of convulsions, without sufficiently discriminating the diseases on which they primarily depend; and even with regard to parents, although alarmed at the first attack, it is astonishing how soon they become in a great measure reconciled to their occurrence. By this means, attacks of convulsions are suffered to become habitual, and a morbid sensibility is generated, which often lasts through life.

Popular belief, as well as the authority of various medical writers, would lead us to suppose that children with large heads are more liable to convulsions than others. This certainly is not in conformity with our own observations; unless the head has increased in size so rapidly as to lose its due proportion to the rest of the body. This, however, is rather to be regarded as constituting chronic hydrocephalus, than simply what is understood by a large head. Our author expresses no direct opinion relative to this point, but says that he has seen convulsions "occur very frequently in children with small heads."

With regard to the convulsions themselves, we are told—

"The parts most commonly the seat of convulsions are the eyes, the features of the face, the superior and inferior extremities, and the respiratory muscles. It perhaps never happens that the features retain their natural tranquillity of expression while other parts of the body are convulsed, although the preternatural movements of the face are sometimes slight.

"Each part may be separately and successively affected, or the whole frame may be convulsed at the same moment. The convulsive movements are sometimes confined to one side of the body. This variety does not, however, alter the nature of the disease, and is not of sufficient importance to warrant the distinction which has been adopted by some authors, of general and partial convulsions. They are each produced by the same causes, and require the same general treatment.

"Neither fever nor disturbance of the intellectual functions forms a part of the symptoms of a paroxysm of simple convulsions. A child may not be able to hear during the paroxysm; but this is not a proof that its faculties are destroyed. The muscles are no longer obedient to the will. Mr. Thompson, of Whitehaven, has recorded a curious case in the Medical Repository, of loss of speech and hearing in a child eighteen months old, who had been suddenly seized with convulsions: her vivacity remained, and her health was unimpaired. She continued in this state until her sixteenth year, when, after the noise of a public rejoicing, she was observed to recover the sense of hearing, and she soon began to articulate.

"There is much difficulty in establishing clear distinctions between the various species of nervous affections: they pass into each other imperceptibly in many cases, and the line of distinction is scarcely to be defined. There is a strict analogy between epilepsy and simple convulsions; the muscular system is in each disorder affected in a very similar manner. An attack of epilepsy, however, commences and terminates by a state of stupor and dulness, which for a time destroys the senses and intellectual faculties. Such is not the termination of convulsions, properly so called. From whatever cause convulsions may occur in highly irritable children, the attack may run into true epilepsy, idiotism, or paralysis. Epileptic paroxysms also return without any obvious cause, and are frequently periodical. The cause of simple convulsions, on the contrary, may generally be determined. The periodical return of the accessions of many nervous diseases, is a subject of considerable interest, and one which is very little understood. Dr. Pitcairn relates the case of a man of thirty years of age, who, from the age of nine years, experienced each year, in March and September, at the commencement of a new moon, a convulsive attack of the right arm. I have at this moment a case under my care, of a young lady, who has been seen by many practitioners, and amongst others by my friends Messrs. Arnott and Ward, who for three years has been affected with a remarkable convulsive cough, which commences in November without any apparent indisposition. It ceases as suddenly as it begins, but has lasted for several weeks at each attack. In this case there is a trifling distortion of the spine. The head is of a remarkable shape; there is also slight strabismus." (P. 18—21.)

At page 25, it is stated that convulsions rarely appear during the night, owing to the patient being removed from the various stimuli which excite them. This is an important observation, as it is directly opposite to what we observe in true epilepsy; the fits coming on more frequently during the night, or just after waking, than at any other period.

The *proximate cause* of convulsions next comes under consideration, and it is no reproach to the author that he has failed in throwing any additional light on this obscure point of pathology. It is asserted that, in the great majority of cases, convulsions are symptomatic; and in this, as already mentioned, we fully concur. But the second part of the position, that " it might probably be asserted with accuracy that they are never idiopathic," requires some discussion.

"Such certainly has been the result of my own observation, although I am diffident of stating decidedly an opinion in opposition to the many authorities of high respectability who adopt the above division. When the brain is acted upon immediately by any cause, and convulsions supervene, they are considered to be idiopathic; but, in such cases, the convulsive action is secondary to some deviation from the natural condition of the brain, and is therefore, in point of fact, as strictly symptomatic as if it were the consequence of derangement originating in some distant organ, and subsequently reflected upon the brain. From the imperfection of our knowledge, or the carelessness of our inquiries, the derangement of which convulsions are symptomatic no doubt frequently escapes detection, and the attack is looked upon as idiopathic." (P. 29, 30.)

According to this explanation, there certainly can be no such thing as an idiopathic convulsion; for, as the muscles derive their motive powers from the brain and nerves, so it is obvious that "some deviation from the natural condition" must invariably precede their supervention. We apprehend, however, that, when we use the terms symptomatic or sympathetic, we always imply a distinct relation to some other disease, and not that "deviation from the natural condition" which proves the immediate forerunner of the convulsions, and which may be regarded as their proximate cause.

It is stated, as an undoubted fact, that the convulsions of children are much more frequent now than they have been at any former period; an assertion, by the by, which is made with regard to so many complaints, that we sometimes wonder how our ancestors ever came to fall sick and die. However this may be, we quite agree with the author in the following remarks on the impropriety of forcing the minds of children, by early culture, into precocious development, as is too much the fashion at the present day.

"We should operate upon the tender intellect of a child by the gentlest progression. It must surely be much more judicious to complete the instrument previous to its use, than to employ it in an imperfect state. It is the same with children as adults. In the cultivation of the mental powers, we are always to bear in mind the capability of the individual to answer the demands which are made upon him for exertion. It is not only irrational, but it is frequently destructive, to impose either upon the mind or body, but particularly upon the former, a load which it is incapable of supporting. It may be a source of consolation to those parents who are too apt to lament any apparent loss of time in the very early periods of life, to remember that early acquirements are not to be gained without destruction of health, and that the future progress and mental powers of the individual depend upon the foundation which is laid in infancy, by judiciously adapting the studies of the child to its age and constitution. By premature efforts to improve the powers of the intellect, the organ in which they reside is exhausted. The practitioner, then, cannot too forcibly reprobate the pernicious enforcement of precocious studies. The injurious effects arising from the folly and false vanity of parents, who are ambitious of holding forth their children as specimens of extraordinary talent, are constantly presenting themselves to our view in a train of nervous symptoms, and of susceptibility to ordinary impressions, which frequently pave the way to decided paroxysms of convulsions." (P. 32—34.)

This injudicious system of keeping young people too long under the restraints required for their premature learning, seems to be an evil felt of old, and to which M. DE SEVIGNE probably alluded when she made the quaint observation—" Je suis persuadée que la plupart des maux viennent d'avoir le cul sur selle."

The following quotation will put our readers in possession of some of the most important of the author's views:—

" Both in children and adults, the effects which arise from any given irritation will depend upon the particular constitution and temperament of the individual. In one, local pain, unconnected with general disturbance, may ensue; in a second, an attack of fever may arise; and in a third, convulsions with or without pain or febrile movement. With whatever train of symptoms an infantile disease commences, or in whatever part of the body derangement of function or disease of structure may primarily be situated, the irritation endured by that part may be reflected upon the brain, and convulsions may follow as a symptom of the cerebral reaction upon the muscular structure. The probability of the occurrence of convulsions will be determined by attention to the particular constitution of the patient. The character of the original disease may vanish, and the treatment at first required must be changed for one more directly appropriate to the transition which has taken place. But, although we are to bear steadily in mind the possibility, and even probability, of convulsions being produced by some serious cerebral disease, it is equally important that we should not hastily determine that the brain is organically, or even functionally, affected, because a paroxysm of convulsions occurs, or prognosticate the speedy effusion of water in the ventricles, unless enormous doses of calomel are prescribed, and repeated bleedings are had recourse to. Such a mode of practice has been recommended by high authority in several cases of convulsions, which I have watched with much attention; and at the same time it has been confidently asserted, that hydrocephalus would ensue if any part of the plan of treatment were omitted. A much more moderate, and I think more rational, plan has been adopted, and the children have perfectly recovered. Unless they arise from mechanical violence or intense moral impressions, idiopathic affections of the brain are, I conceive, very rare in children. I by no means agree with the opinion expressed by the French physicians,* that, when convulsions are symptomatic of encephalic inflammations, it is almost always the case that some evident cause has been applied, which has acted mediately or immediately upon the head, and from which inflammation almost certainly arises,—such as blows, violent concussions, falls, &c. &c. Convulsions quite as frequently occur during inflammation of the brain, which is produced by sympathy with some distant part that is highly irritated. It is also said by the same authority, that there is a well-marked difference in the convulsions themselves, when they arise from affections of the head. It appears to me, when a paroxysm of simple convulsions is once excited, whatever may have been the cause of it, that it is essentially the same, although it may differ much in its degree of violence and duration.

"It is a frequent and a fatal error in the practice of the present day, to seize upon some individual symptom, such as slight strabismus, partial convulsions, or a crouping noise in the breathing, and to determine that water in the head will inevitably be the consequence, unless a particular and formidable treatment be employed. Each and all these symptoms may, however, arise where we have no reason to suspect any affection of the brain." (P. 47—51.)

It is a very common idea that the desiccation of cutaneous discharges proves a frequent source of convulsions: probably this opinion has been carried somewhat too far, even in this country; and in Germany, the importance attached to it is most extravagant, repressed, or imperfectly developed; itch being held by some in that country to be the immediate cause of almost all diseases under heaven. The author before us "doubts" (and we participate the doubt,) whether convulsions are ever produced by the natural or artificial disappearance of eruption, provided a moderate action has been kept upon the bowels. But we cannot go along with him in what follows, although he is backed by the authority of most writers. We allude to the generally received doctrine, that occurrence of convulsions on the supervention of exanthematous fever, particularly small-pox, was a favourable omen. "From my own experience, (says Mr. North,) I should infer that this opinion is well founded, provided the eruption makes its appearance at the usual period from the commencement of the premonitory symptoms." All our own experience is opposed to this, and we have learnt to look upon convulsions, under the circumstances alluded to, as indicative of a very severe disease; and all we can venture to say is, that a convulsion fit previous to, and followed by, the eruption of an exanthem is (*cæteris paribus*) not so dangerous as a fit from any less obvious or more perma-

* Rapport sur les Prix xxxi. prefixed to Brachet's Mémoire sur les Convulsions des Enfans.—Paris, 1824.

nent cause. Baumes, in his work "sur les Convulsions dans l'Enfance," lays down the occurrence of convulsions previous to the supervention of an eruptive disease, as justifying the prognosis of a fatal termination.

Among the alleged causes of convulsions, almost every writer has enumerated worms; but we agree with Mr. North in regarding this as a much less frequent cause than is generally imagined: he states that he does not remember a single instance where convulsions appeared to depend upon the presence of worms in the intestines, or to be relieved by their expulsion.

Leaving the causes, we now come to the *symptoms;* and any one who has been accustomed to see much of the diseases of children, will recognise the fidelity of the following picture:

"From whatever cause a great predisposition to convulsive affections exists in children, the following symptoms are usually characteristic of that state of increased irritability from which their occurrence is to be anticipated. Although it cannot be said with truth that the child is ill, he is evidently threatened with disease. It will be observable, that during the day he starts with apprehension at the most trifling noise. His sleep is disturbed with sudden cries. Not unfrequently he sleeps throughout the day, and remains restless and entirely sleepless during the night. Whatever might have been the natural placidity of his temper, he now becomes peevish and irritable; quarrels with his companions; and derives either no pleasure at all, or but a momentary amusement, from his most favourite playthings, which will be suddenly thrust away after having slightly occupied his attention. The eyes are frequently fixed, without being apparently directed to any particular object; or they are thrown upwards, and are stedfastly fixed upon the ceiling. The pupil of the eye is sometimes for a moment contracted, and then suddenly dilated. I have frequently held a candle close to the eye of a child, when I have anticipated the occurrence of convulsions, in order to remark the effect produced. In some instances, where the pupil has been contracted, at the moment the light was applied it has suddenly dilated, and as suddenly again contracted, the light being steadily held close to the eye. The effect of light upon both pupils is not always similar. One may remain fully dilated, while the other contracts; or one pupil may remain stationary, the other being alternately contracted and dilated. I am not aware that the remark has been made before; but I believe, from frequent observation, that when a light is applied close to the eyes, and the same effect is not produced upon both pupils, that we have much reason to fear some serious affection of the head. It is now, I believe, generally admitted that the mere dilatation or contraction of the pupils is dependent upon so many and dissimilar deviations from health, that no particular inference can be drawn from either of these conditions of it. An oscillatory motion of the pupil is very frequently one of the indicative symptoms of approaching convulsions.

"It is always useful in diseases to examine the position of the limbs during sleep, particularly the sleep of children. If they deviate from the ordinary degree of flexure to the more straight position, there is generally some irregularity in the state of tone, and of course in the vital influx. Upon viewing the position of a child during sleep, whom, from the occurrence of symptoms above mentioned, we consider disposed to convulsions, we shall frequently find the limbs almost rigidly extended, the great toes and thumbs being turned inwards. Stretching of the limbs, it is true, is both in adults and children a natural action, which is exerted to restore muscular equilibrium. In connexion with several of the other premonitory symptoms, however, it must be considered as a strong indication of a tendency to convulsive movements.* The colour of the countenance varies frequently in children strongly predisposed to convulsive paroxysms: at one moment it is pale, at another highly flushed. No corresponding variation of the temperature of the surface of the body is to be detected. For a short time the countenance of the child is expressive of great animation; the eyes are vivid and glassy in appearance. Suddenly, and without cause, he appears languid and inclined to sleep. The breathing is irregular. The child frequently draws long and deep inspirations with apparent difficulty, and these are alternated with a short and catchy breathing. This disordered respiration I consider to be peculiarly indicative of approaching convulsions. It is usually accompanied by a fulness of the upper lip and a contracted appearance of the nose, which alter the natural expression of the countenance. The hands are frequently directed towards the nostrils, apparently without any voluntary effort. If we observe the fingers of a child highly disposed to convulsive diseases, we shall see them either in frequent and sudden motion, or firmly pressed towards the palm of the hand. The thumb is more frequently contracted upon the palm, the fingers at the same time being extended and separated from each other. In a discussion which lately took place at a medical society upon this subject, it was contended, by an accoucheur of much celebrity, that this contraction of the thumb is not to be regarded as a premonitory symptom of convulsions. From my own observation, however, I can state that, in children in whom we detect this firm contraction of the thumb, convulsions will almost certainly occur at some subsequent period; unless, indeed, the suspicion of the practitioner is roused, and appropriate remedies ward off the threatened attack. When the child is put to the breast, it sucks eagerly for a moment, and ceases suddenly, throwing back the head with an expression of anxiety in its countenance, and perhaps rolling it from side to side. Deglutition appears to be performed with difficulty when these symptoms occur." (P. 69—75.)

The injurious effects of over-feeding are fully and forcibly exposed: "so long as nurses and mothers believe that children thrive in proportion to the quantity they eat, so long will convulsive diseases be frequent and severe." There can be no possible question—at least with any practitioner of common

* Good's Study of Medicine, vol. iii. p. 303.

sagacity—that children ought to be fed on plain unstimulating food, while they ought never to be permitted to overload the stomach even with this; and that, under ordinary circumstances, the breast of the mother ought to afford the exclusive nourishment of infants. Where, however, the mother is prevented from suckling, the greatest care is required in choosing a substitute; and numerous instances are alluded to by Mr. North, of convulsions and other serious injury resulting to the child from the unhealthiness of the nurse.

The improper use of certain patent medicines, containing opium or some other narcotic, is pointed out, and no doubt with justice, as favouring the occurrence of convulsions.

It is maintained by some, that dentition, being a natural process, cannot with propriety be enumerated among diseases. However this may be, no one who knows much about children will deny that the process frequently gives rise to various diseased actions. In this respect it is precisely on the same footing as parturition, which, though a "natural process," proves the exciting cause of many inconveniences, and not a few morbid affections.

"It was supposed by the ancient physicians, and the same opinion is supported by a few modern authorities, that children never die from the effects of difficult dentition. If we allow, with Wickmann and others, who have urged the impossibility of disturbance to the system from painful dentition, that the gums are senseless, or provided with no particular sensibility, we are not compelled to grant that these parts are deprived of sensation during dentition. In health, bones and cartilages possess little or no sensibility. In disease, they become exquisitely painful; and, although the process of dentition cannot be considered in every case a disease, it is subject, like every other natural operation, to accidental disturbance, which will be productive of local and general suffering. In Germany it is a current opinion that children rarely, if ever, suffer from the effects of dentition. The disturbance which so frequently occurs during the progress of dentition, is attributed by the practitioners of that country to other causes: hence they rarely, if ever, advise division of the gums.* Many children must undoubtedly be the victims of so erroneous an opinion. It is difficult, indeed, to reconcile the maintenance of such a doctrine with the character which the German practitioners bear for the accurate investigation of the causes and symptoms of derangements of health, and for the industrious zeal with which they add to their own stores of information from foreign sources. That children do suffer considerable distress during the process of dentition, is certain; and that the pressure of the tooth upon the nerves of the part is frequently the cause of the severe train of symptoms and convulsive paroxysms under which they labour, must be

* Wickmann, Ideen zur Diagnostik. Hecker, Magazin für die Pathologische Anatomie.

equally clear, from the almost instantaneous cessation of suffering when the gum is freely divided. It cannot be doubted that, if assistance were not afforded by this simple but effectual operation, children would frequently sink under the consequences of painful dentition." (P. 108, 110.)

Among the appearances found after death, vascular turgescence at the roots of the nerves, effusions, inflammations, &c. &c. are enumerated. Sometimes these have been found in the cerebrum or cerebellum, sometimes in the spinal cord; but it is observed, that it may be doubted whether these appearances may fairly be regarded as the causes or effects of the convulsive paroxysms.

There are some good remarks relative to *prognosis*, which we shall give at length:—

"It is frequently difficult to determine either the duration of a convulsive paroxysm, or the ultimate consequence of its frequent repetition; and we shall therefore be rarely justified in giving a certain prognosis. Sometimes the most violent and frightful paroxysms pass off without inflicting any serious injury, either upon the present or future health of the child. In other instances the attack, apparently slight at its commencement, either destroys the child in a few moments, or paves the way for its subsequent death, by entirely destroying the powers of mind and body. It follows, then, that, although there are no diseases more likely to excite terror than convulsive affections, the danger is not to be estimated by the apparent violence of the attack. Our prognosis must not be positively guided by the external appearances or degree of the convulsive movements. We must consult rather the cause of the convulsions, and the nature of the disease of which they are symptomatic. The constitution of the child must also be considered. The danger is much less in a child of great susceptibility, than in a robust subject, who is less easily excited. In the former, convulsions will occur more readily: in the latter, they will be infinitely more severe. The younger the child, the less is the danger we are to apprehend from convulsions. In very early infancy, an equal degree of danger attends the convulsive affections of both sexes. After that period, girls are more subject to convulsions, and less seriously affected by them, than boys. In children of a sanguine temperament, the attack is commonly violent, and passes quickly to a termination, whether it be fatal or not. In the opinion of Brachet, convulsions arising from external and mechanical causes are less severe, unless some important organ has been injured, which accident in itself constitutes the danger. My own experience would induce me to give a contrary opinion.

"Convulsions which arise from irregularities of diet, are generally severe and hazardous. If convulsions are caused by exposure to cold, their degree of severity and the danger will depend upon the extent to which the brain is affected. Congestion in the vascular system of the head is much to be feared in such cases. If any sudden moral emotion is productive of convulsions in a child, the attack is in general slight and transient. I have mentioned, at page 85, one fatal case arising from fear. Convulsions occurring during the course of any severe disease, strongly indicate its danger. Recovery is very doubtful if the intellectual functions suffer. "*Sensus etiam suppressi majus periculum in omni spasmorum genere, quàm integri ostendunt.*" (VOGEL.) Partial are less dangerous than general convulsions. The more frequent the return of the convulsive attack, the greater is the probability of a fatal termination. It is a well-known fact, that convulsions are infinitely more common and more dangerous in hot climates than in the more temperate regions. Cases occasionally occur in which convulsions appear to mitigate the severity of any existing disease. Brachet very justly ridicules the endeavour of Sauvage to prove that convulsions are always beneficial.

"We have yet to learn how to determine positively when convulsions do arise, and when they do not, from organic lesion of the brain, or from effusion of water into its ventricles. When the convulsive attacks are slight and of short duration, and are succeeded by the natural cheerfulness of the child, we have but little reason to apprehend any danger. On the contrary, when the paroxysms are of long continuance, and gradually increase in severity and violence, and leave the child dull and heavy, we have much cause to apprehend a repetition of the attack, and should give a guarded opinion as to the ultimate consequences. In all cases which I have myself witnessed, where the child was destroyed suddenly during convulsions, the dark colour of the face and neck, and the almost stertorous breathing, indicated a state very nearly allied to apoplexy in the adult. I have unfortunately not been permitted to verify the accuracy of this supposition by dissection: that it is well founded, however, I think there can be but little doubt. Death may occur also in consequence of the respiration being impeded by the irregular contractions of the respiratory muscles. The lungs become engorged with blood, and the circulation through them is impeded. Suffocation is quickly threatened, and destroys the patient, unless the natural action of the muscles is restored, and the respiration and circulation are enabled to proceed without interruption. In some cases a state of syncope supervenes to convulsions, from which the child never rallies. It is much to be apprehended that in some cases where a state of syncope has supervened to convulsive paroxysms, that most horrible of all accidents has occurred—premature interment. However sceptical we may be as to the truth of many of the cases which are recorded, it must be admitted that there are some instances, which rest upon very credible authority, of women and children who have very narrowly escaped being consigned to the grave before the vital spark was extinct." (P. 124—130.)

*Treatment of convulsions.*—As the causes which excite convulsions are both numerous and dissimilar, it is obvious that the treatment cannot be always the same, but must vary with varying circumstances. During *the fit*, indeed, but little can be done, and that little is had recourse to rather with a view to satisfy the friends and gain time, than from the expectation of its producing any very decided effect. The warm bath is generally recommended, and it is in most cases the best mean we can employ. With this may be joined frictions on the chest, belly, and spine, with some stimulating and anodyne liniment; and a purgative glyster may be thrown into the rectum. All attempts during the paroxysm to force the child to swallow medicines, are for the most part unavailing, and frequently injurious: as a general rule, they ought to be avoided. It becomes a question, however, of interest, and it must be confessed of some difficulty, to determine whether we ought or ought not to abstract blood. Mr. North informs us that, "if there are evident marks of determination to the head, blood should be drawn from the jugular vein;" but, on the other hand, we are cautioned against taking away blood during a convulsive fit, merely with the intention of relieving its violence.

Dr. Brown, of New Orleans, has advised the application of gradually increased pressure upon the stomach, or of a light roller round the abdomen. We have no experience of this ourselves, nor does the author do more than mention it.

Our readers may remember that Dr. Currie speaks very highly of the effects of the cold bath; but his recommendation has never been generally adopted. It will be kept in mind that we speak of the treatment *during* the paroxysm.

In this, as in every other disease, it is a matter of the first moment to detect the cause, that our treatment may be regulated by rational principles. "If the child is robust and of a plethoric constitution, with the head hot and disproportionately large, the carotids throbbing, the countenance flushed, the eyes sparkling and projecting from the orbit; if he sinks into a state nearly approaching to coma, after any unusual agitation, we have reason to fear a hazardous determination of blood to the head, and must proceed accordingly." (P. 145.) By proceeding "accordingly," however, the author does not mean that we should adopt the usual practice of putting leeches to the temples. "I must be allowed to declare my utter want of confidence in the practice which is recommended by almost every authority. I allude to the application of a few leeches to the temples. I have never seen well-marked symptoms of determination of blood to the head in children removed by leeches, however freely they were applied." (P. 146.) We cannot agree with the author in this doctrine: in what is called *determination*, we suppose the diseased action to be short of inflammation, and we have found the application of leeches at once the most simple and most effectual method of affording relief.

Mr. North, however, prefers opening the jugular vein, or cupping behind the ears; and that these produce more speedy depletion, and are therefore to be preferred in inflammatory attacks, there can be no doubt.

The author does not speak favourably of bleeding in the arm; and, not content with expressing his own disapprobation of the measure, he indulges in a gibe at those who recommend it:—"That physicians should speak of the performance of the operation with confidence, is not a matter of astonishment. Their part of the duty is not difficult; they order, but have not to act." (P. 148.) Now, we are quite aware that this, like many other things, is "easier said than done," and that the operation is attended with more difficulty than in the adult; at the same time, that it may often be practised with success, admits not of doubt. We ourselves question whether venesection possesses any decided advantage over cupping; but, if the physician thinks bleeding from the arm a measure likely to be of use, it is his business to order it, and it rests with the surgeon to say whether it is or is not practicable in any given case.

The quantity of blood to be taken must depend entirely upon circumstances, and the author does not attempt to lay down any "precise and dogmatic rules." After bleeding of any kind, the child is to be kept as quiet as possible, to avoid the nervous irritation which is apt to supervene.

The bowels, as a matter of course, are to be kept open, and this may best be done by "proper doses of calomel combined with jalap." But the author "entirely disapproves" of those large and frequently repeated doses of calomel which are so often had recourse to, and to which the name of *heroic* has most absurdly been applied.

Cold applications to the head are much commended, and the necessity of taking care that such applications are *bona fide* cold, and not consisting "of a damp rag which has perhaps been intentionally *warmed* by the hands of the over-officious nurse," is very properly insisted on.

Blisters are next spoken of; and, as the opinions expressed are very different from those generally entertained, we shall lay them before our readers.

"I am far from partial to the application of blisters to children; but I apprehend that the principle of counter-irritation, upon which they have been recommended to be applied to the lower extremities, has been unjustly ridiculed. In many cases where there was evident determination of blood to the head, without any general excitement, (which is a state we constantly observe in children,) I have seen the best effects produced by following the advice of Dr. John Clarke, and applying blisters to the calves of the legs or between the shoulders. Blisters to the head are decidedly prejudicial, although they are not unfrequently applied by practitioners of experience. They certainly keep up a discharge from the integuments of the head; but this effect can only be produced by increasing the arterial action within the cranium." (P. 158.) [illegible]

In this paragraph it will be seen the author expresses himself against blisters, except under certain restrictions; but, in another place, he indulges in a regular philippic against them.

"If I may venture to express an opinion which has been impressed upon me by repeated observation, notwithstanding it is in direct opposition to all the doctrines I have heard maintained in the medical schools, I should say that, if blisters were never applied to children in any case whatever, much less evil would arise from the want of them, than is in common practice daily, or perhaps hourly, inflicted by this popular and painful practice. What can be more unjustifiable than the language which we constantly hear employed upon the subject of blistering children. It is in the mouth of every old woman, and of some practitioners too, that 'a blister is a fine remedy, and that at least it can do no harm.' Is the infliction of many hours' torment no harm? When a remedy is evidently applied without any settled principle, it is a subject of fair inquiry to investigate the claim it has to our confidence. If a child is in a state of coma from presumed oppression of the brain, and if the practitioner wishes to excite every organ to increased activity, and to rouse the nearly extinguished powers of life, he applies a blister, and perhaps with benefit. This is the only condition in which we can look with any degree of reliance upon blisters in infantile diseases, and in which we need not be apprehensive of any bad effects. But the very same practitioner, if he has to treat a case of local inflammation,—pneumonia, for example,—will seek assistance from the same remedy. The disease itself is productive of much general irritation, and of considerable local distress. Whatever is likely to act as a stimulus must be prejudicial, although we may be obedient to the instructions of allaying the severity of the attack by bleeding, &c. before we have recourse to blisters. In the latter case, the condition of the patient is totally the reverse of the former. If, in the comatose state, a blister acts beneficially as an excitant, it must be prejudicial in the other, for the same reason. I confess I should leave entirely out of the question the benefit it is presumed we derive from the counter-irritative effects of blisters, when applied to young children. Excepting in the particular cases I have referred to, I believe with much confidence that the advantage from blistering is rarely equivalent to the pain and general irritation it produces. The period at which we apply blisters in local inflammatory affections, is not to be forgotten. We first subdue the severity of the disease by other and appropriate remedies, and, when it is upon its decline,—when, in all probability, the unassisted powers of nature would successfully perform the remainder of the task,—a blister is applied. The patient gets well, notwithstanding the additional pain thus inflicted; and the fortunate result of the case, which is really to be attributed to the measures previously employed, is said to be owing to the good effects of counter-irritation, &c., and the blister gains a character to which, in point of fact, it has no claim." (P. 202—206.)

It is acknowledged that, if a child is in a state of coma, a blister may be of service; but it is asserted that in a local inflammation, pneumonia for example, the same remedy will be applied; whereas "if, in the comatose state, a blister acts beneficially as an excitant, it must be prejudicial in the other for the same reason." We would remark with respect to the coma, that, in the great majority of cases, it is the result of increased vascular action which has ended in effusion, the reabsorption of which frequently appears to be promoted by the application of blisters. With regard to the pneumonia, it certainly is not customary, with practitioners of any experience, to have recourse to these remedies until the inflammation is on the decline; and, as effusion into some of the pulmonary textures is the most frequent termination of this disease, by which the functions of the lungs become impeded, so it appears to us that we employ blisters for the same purpose in both cases; viz. to remove those effusions which are apt to follow increased vascular action, whether this may have been confined to simple determination of blood, or passed into actual inflammation; we would, therefore, be disposed to reverse the proposition of the author, and to say, that "if in the comatose state a blister acts beneficially as an excitant, it must be *of use* in the other for the same reason." We do not speak of the effects of blistering children without some experience, and we have no hesitation in giving it as our opinion that, in inflammation of the chest, as soon as there is evidence of the increased action relieving itself by effusion, blistering becomes a powerful auxiliary. At the same time it is to be kept in mind, that the greater vascularity of the cutis in children renders the stimulus of the blister necessary for a much shorter time, and that, as a general rule, the plaster ought to be removed as soon as any distinct redness is produced: this, in children under three years old, generally takes place in three hours, and scarcely ever requires more than five. By attending to these restrictions, we have never, in our own practice, seen sloughing produced; and, as the patients generally got well, we have as much right to suppose that their recovery was promoted by the blisters, as Mr. North can have in attributing it to "the measures previously employed."

When, however, we have had recourse to depletion in the usual way,—that is, when we have abstracted a quantity of blood proportionate to the age of the child; when we have acted upon the bowels, and enforced a rigorous diet; we shall find, notwithstanding, that a return of the convulsions will frequently take place: the increased vascular action and local determination have given way to a state of general irritation; a state, to obviate which very different remedies are required.

"The child will be sleepless, exceedingly fretful, the pulse rapid and small, and slight twitchings of different muscles and tendons will be detected, if strict attention is paid. For a moment or two the carotids will beat violently, but this increased force will suddenly be followed by a very languid action of them. The countenance is generally pale and distressed, and the brows are wrinkled. Pain in the head is rarely complained of, if the child is old enough to express its feelings. In this state further depletion is certainly not demanded, although it is frequently practised: it will not only be unnecessary, but absolutely prejudicial. The severity of the symptoms will increase in an equal ratio with the employment of debilitating measures. What are the means, then, which we are called upon to pursue under such circumstances? In my opinion, the exhibition of sedative medicines is imperiously demanded; and I believe small doses of the pulv. ipecac. comp. is the best remedy. I have frequently given the extract of hemlock or henbane, in conjunction with alkalies, with much advantage. These medicines, however, sometimes fail to relieve the irritable state above mentioned, when Dover's powder will succeed." (P. 162—164.)

The third and fourth chapters treat of *Infantile Epilepsy*. Into neither of these will our limits permit us to enter: they contain a very fair view of all that is known upon the subject, but the author has no new theory to bring forward, while he has had too much experience to vaunt any remedies as infallible.

The fifth and sixth chapters, which complete the volume, relate to a *spasmodic affection of the chest and larynx* in young children, accompanied by general or partial convulsions. This is a condition which, we believe, is frequently confounded with croup, from the shrill and sonorous respiration which accompanies it; and we know of an instance in which a nurse has gained credit for having cured a child of the croup five times, by means of the warm bath and castor-oil. Our readers will find the original papers of Mr. North upon this subject in the fifty-third Volume of this Journal, pages 39 and 284, which would render it a work of supererogation for us to resume the discussion.

We may be permitted to remark, however, that it is by no means the least interesting part of the volume; and that, in our opinion, he satisfactorily establishes that this crouping noise in the throat, with drawing inwards of the thumb, &c. is not necessarily connected with any organic disease in the head; and, consequently, does not require the same degree of activity in the treatment which this view of its pathology would demand.

### DIVISION II.

### FOREIGN.

ART. II.—*Traité Elementaire de Diagnostic, de Prognostic, d'Indications Therapeutiques; ou Cours de Medicine Clinique.* Par M. L. ROSTAN, Medicin de l'Hospice de la Viellesse (Femmes), ci-devant Salpetriere; Professeur de Medecine Clinique, &c. Tome premier.—Paris: chez Beschet, jeune, Libraire. 1826.

*An Elementary Treatise on Diagnosis, Prognosis, and Therapeutical Indications; or a Course of Clinical Medicine.* By M. L. ROSTAN, senior Physician to the Salpetriere; Professor of Clinical Medicine, &c.—Paris: Beschet, the younger. 8vo. pp. 587.

THIS book, although, from its elementary nature, it is not adapted for critical analysis, nevertheless contains too many valuable practical remarks, the result of attentive observation and considerable experience, to permit us to pass it by in silence. It is not ushered into the world by any preface or advertisement whatever; nor are we even informed to what length the whole work is likely to extend. It would seem as if M. ROSTAN considered the sanction of his name as quite sufficient to fix the attention of the profession to his performance; that he was under no necessity to explain his motives or design to his readers; and still less to make any apologies for omissions or imperfections, which indeed are but too often mere words of course.

It is not our intention to go through this volume page by page: it is sufficient for our purpose to say that the first part is entitled *Proligomena*, and contains general remarks upon the organisation of functions of the living animal in health and disease; upon the utility of clinical medicine, the advantages of pathological anatomy, the object of medicine, and other matters purely elementary. This first *part* (which term our author employs in the sense usually applied to the word *chapter*,) occupies ninety-six pages.

The second part treats of *Diagnosis*, and commences with these general considerations:—

"Independently of the knowledge of man in a healthy state, three things are indispensable in order to form a just diagnosis:—1st. A knowledge of those changes which take place in the functions or organs, and this constitutes *symptomatology*. 2dly. The proper appreciation of each of these phenomena; in other words, the knowledge of what they *signify:* this is *semiology*. And 3dly. A knowledge of the characters of the different diseases which afflict humanity; of the features which distinguish one from the other: this is especially called *diagnosis*."

The greater part of this chapter is made up of arguments to prove the necessity of attending to pathological anatomy, and the absurdity, or worse than absurdity, of prescribing merely from symptoms. Of the mode in which our author treats this part of his subject, we shall offer the two following examples:

"Do you wish to have (says M. Rostan,) a striking and novel example of the vicious nature of this plan? [*la medecine des symptomes,*] we shall find it in palsy. It is not long since we were ignorant, and many seem yet not to know, that palsy is merely a sign of some alteration in the brain or its appendages. Pathological anatomy has placed this beyond all doubt. Well, then, what was the practice? They rubbed and inflamed the affected limb, in order to bring back its feeling or function. If this was of no use, at least it was harmless. But at length somebody thought of giving nux vomica to an animal, and it was then observed that the hinder limbs were affected with convulsions, and thence it was concluded that this remedy would work miracles in cases of paraplegia commonly called nervous. Soon afterwards it was given in hemiplegia: it was observed to produce convulsions in the paralysed limbs, and therefore it was supposed that it would restore their motion. If these physicians had but known that these symptoms were only the effects of a local disease of the brain, either acute or chronic, they would have seen that the method proposed of agitating the affected limbs, could only do so by acting upon the diseased part of the brain, and therefore would rather hinder than facilitate a cure. They would have seen that exciting the motion of limbs in such a case, was exactly parallel to that of rubbing the extremities of broken bones against each other, in the hope of consolidating a fracture. They would also have seen that a palsy depends upon eight or ten different diseases, and therefore it was absurd to combat them by the same means." (P. 111.)

Take another example:—

"The efficacy of prussic acid in consumption has been proclaimed. One would have believed that these authors have thought that pulmonary consumption was always one and the same disease, or that they had determined in what particular species the prussic acid was available. Not at all. Whatever the species of phthisis was, the prussic acid was always excellent, and the servile crowd of imitators continued to extol and employ it."

From the first chapter on *Symptomatology*, we shall make but few extracts, since the greater part consists merely of an enumeration of the functions deranged, and the mode in which they are found to be so. We, however, shall translate the introductory passage, and then proceed more at large to discuss the *signs* deducible from each of these symptoms in succession.

"Every morbid change (observes M. Rostan,) which takes place in the organisation is called a *symptom:* these changes manifest themselves both in the organs and in the functions which they execute. From an alteration of function, we may conclude that there is a change in the organ to which the function is confided. When by this means the condition of the deranged organ is recognised, as well as what the lesion is, the *symptom* becomes converted into a *sign*. This phenomenon, having led us to recognise the disease, has acquired a *meaning*. Our senses alone enable us to ascertain symptoms; in fact, they are equally apparent to those unacquainted with the art of medicine: all the world knows what a pain in the side is, but it is only the physician who is aware of its *signification*. The changes which our senses make us acquainted with in disease are, in the organs, either in their augmentation or diminution, (*hypertrophy, atrophy*,) their consistence or temperature, alterations of form, position, colour, smell, and sound. The complete abolition of an organ is rarely an effect of disease. With regard to the function itself, these changes may be reduced to augmentation, diminution, perversion, or complete abolition. The results of the functions,—that is, the matter secreted and excreted, also demand the attention of the physician. Regard must also be had to their augmentation, diminution, perversion, and abolition. The whole of symptomatology is included in this short exposé, and there only remains to make the application to all organs and functions in a physiological order." (P. 122.)

This, as we before said, is the object of the remaining pages of this chapter, commencing with the organs of digestion, and ending with those of generation. The fourth section, however, becomes of practical utility, and we shall resume our extracts from thence.

" Nothing is, perhaps, more embarrassing (we quote M. Rostan's words,) to a young physician who has just begun to exercise his profession, than the mode of examining and interrogating a patient. The first thing which occurs is, of course, an examination of the external state: the physiognomy almost involuntarily fixes the attention in the first place, from which we can judge of the age, strength, and the state of the patient's mind; circumstances of the greatest importance in forming a correct prognosis, and in establishing the indications of cure. This examination must not be confined to the head only, it must extend to all parts of the body; by which we form a judgment of the stature, make, colour, conditions of eruption, marks of wounds, deformities, &c. Unfortunately this scrutiny is necessarily much limited even in hospital practice, and in private life it is impossible, in the majority of instances: however, an attentive examination of a part *in pain* is necessary to enable us to avoid committing serious mistakes."

The following example is given:—An aged female, of weak intellects, was received into the infirmary; she complained of a severe pain in the abdomen, towards the left iliac fossa. The countenance was lively, the pulse strong and frequent, the skin hot and perspiring, the tongue dry, with great thirst; in other respects, the digestive functions were in a healthy state. The pain in the abdomen was aggravated by pressure or motion. M. Rostan gave the following diagnosis:—The phenomena of reaction announce an acute inflammatory state; the local signs show that the abdomen is the seat of the disease; but, as the digestive functions are healthy, it is evident they are not affected. The slightest pressure is painful, therefore the disease is superficial; motion is painful, therefore the motive powers are affected, *the muscles of the abdomen are the seat of the disease;* although it is true that rheumatism in old people does not usually give rise to general symptoms so strongly marked.

Satisfied with this opinion, our author quitted the patient, after prescribing some simple remedies; when a pupil, raising the patient's shift, discovered an eruption of zona, *(herpes zoster?)* This lesson taught M. Rostan the propriety of applying to his senses, as the only means of positive instruction.

M. Rostan is disposed to condemn tedious interrogations, entering into tedious minutiæ, and observes that, in some diseases of the respiratory organs, for example, too much talking is a great evil in itself. The same remark applies, perhaps with more force, to affections of the brain. The first question to be put to a patient is, "Where are you in pain?" for, as patients have always a disposition to give us their own opinion of their complaints, it is very unwise to ask merely, "What is the matter with you?" You may perhaps receive for answer, "I have a nervous complaint." The next question of importance is, "How long have you been ill?" This resolves the disease into acute or chronic, and narrows the circle of after inquiry very much.

Having made ourselves acquainted with the affected function, it is necessary to examine those parts which are either influenced by or exercise an influence over the diseased part; by this means we arrive at the knowledge of sympathies, and we are also enabled to detect concomitant maladies; for it often happens that many diseases exist in the same individual, and therefore we may commit a fatal error if we are contented with having detected one only.

We omit the methods of employing percussion and mediate auscultation in diseases of the chest, as described by M. Rostan. They have recently occupied a considerable share of our attention, and the method of instituting these inquiries is now sufficiently familiar to the profession.

Some remarks upon the method of feeling the pulse next ensue; and here we are warned not to be too precipitate in this part of our examination, since the very arrival of the physician will often have an effect on the circulation.

Many very just observations occur on the subject of investigating the condition of the viscera by pressing upon the abdomen; but there is nothing particularly worthy of extraction either in this portion of the section, or in that which relates to the examination of the mouth and fauces, and the condition of the uterus; and further on the means of detecting feigned diseases, and those in which the patient has either entirely or in part lost the power of detailing his feelings and symptoms.

After having concluded our examination, it still remains to inquire into antecedent circumstances, which have operated as causes of disease: first, whether it is hereditary or acquired; whether it has now shown itself for the first time;—if it has been met with before, what remedies were then employed, and with what effect;—and, finally, the age, sex, constitution, habits, and profession of the patient, must engage our attention.

Diseases do not always assume the same aspect at different times of the day; hence we are taught to make our investigations in the evening as well as the morning, and perhaps also, upon some occasions, in the middle of the day.

Even when death ensues, the labour of the observer is not finished: it is here that we are to search for the correctness of the physician's judgment. The examination of the dead body requires the same care and attention as the living patient: it ought, indeed, to be very scrupulously performed, because, when we have once destroyed the organs, we cannot refer to them again. There is much good sense, as well as candour, in the following paragraph, but, unless we had found it in the work of a Frenchman, we should have scarcely been disposed to give implicit credit to it.

"It must be allowed, that in France we are too rich in materials for instruction. We do not sufficiently appreciate the immense advantages of the examination of the dead body: we perform this operation with nonchalance and carelessness, and are far from drawing all the advantages from it which it is capable of affording. Observe with what eagerness foreigners, who are deprived of these precious researches, proceed to these investigations;—what pains, what minute attention, they bestow upon them; nothing escapes their observation. Do you suppose that Morgagni had any great number of bodies at his disposal? Certainly not; but he drew from those which he was enabled to obtain all the advantages possible; he did not forget to examine every thing with his own eyes, and the result has been the collection of a most precious store of facts."

Now, as we said before, though these remarks apply, perhaps very justly, to the general study of anatomy in France, we think that M. Rostan scarcely does justice to his countrymen, as far at least as pathological anatomy is concerned.

In directing the method of examining the dead body, our author follows the plan laid down by M. Chomel, of commencing with the abdomen; which is especially proper if there be any suspicion of effusion or extravasation of any kind within that cavity. He directs us to reserve the examination of the spinal marrow to the last.

The physician is not only occasionally called upon to interrogate his patient, but he must take notes of the case, either with a view of after consultation, or in the event of his wishing to publish it. Now, the art of recording our observations is not a very easy one, according to our author; for he requires the observer not only to possess a profound knowledge of his art, but that he should also be provided with sagacity, be capable of close attention,—and that he should be likewise endowed with sensibility, taste, and even imagination. M. Rostan, conceiving that the necessity of these last attributes may perhaps astonish his readers, thus explains his meaning:—

"A cold observer, an impassive spectator of the ills of humanity, may perhaps be an exact observer, but, if he be not endowed with sensibility, touched with the sight of these evils, is it likely that he will describe what he sees with that warmth which gives a life and soul to description? Whence comes it that we perceive such a marked difference between the observations of different physicians? Whence does it happen that one overwhelms us with fatigue, whilst we peruse the other with the liveliest interest? Is it not because the one is totally void of taste, imagination, and sensibility." (P. 230.)

This section concludes with a table, minutely pointing out all the circumstances to be considered in examining into the state of a patient; though our author very properly observes, that it is not necessary to go through the whole of these researches in every individual case. We should hope not, indeed; for the inquiries comprise every part and portion of the body, and amount to upwards of two hundred.

The second chapter occupies the whole remaining portion of the volume, and treats of *Semiology*. The art of converting symptoms into signs, is, says M. Rostan, without contradiction the most difficult part of the science of medicine. This art our author thinks has made great progress within the last ten years, and, among those who have contributed to its improvement, he especially mentions Laennec, Broussais, and Landré Beauvais. Indeed, throughout the work, our author very carefully confines his quotations and praises to his own countrymen, and appears to be perfectly convinced that the science is no where else so well understood.

Signs have been divided into *past*, *present*, and *future*. This division does not appear, however, to M. Rostan to be deserving of being preserved; and his objection applies to the first term of this division, to which, for obvious reasons, he prefers the name of *commemorative signs*, and they include the age, sex, constitution, habits of life, idiosyncracies, antecedent diseases, remedies that have been previously employed, &c. &c. The meaning and propriety of the terms *diagnostic* and *prognostic signs* is sufficiently obvious; but, says our author, there is a great omission in all works on Semiology, which is that of *therapeutic signs;* for, a morbid phenomenon indicates that such a particular remedy ought to be employed, it becomes therefore an indication, or, in other words, a therapeutic sign. Diagnostic signs have been divided into common and proper; the latter are otherwise called *pathognomic:* these are, of course, the most valuable.

Those signs which M. Rostan calls *local* belong to this class. The former, which are more equivocal, belong to a great number of diseases. Those phenomena which occur during the course of a disease, but which are not essential to it, our author terms *accidents.* There are but few signs, observes M. Rostan, that are truly pathognomic; therefore a sign, however strongly marked, has but little value, unless fortified by others. Thus, a pain in the side is equally appertaining to any disease of the chest; but, if the patient at the same time has a bloody expectoration, the probability of his labouring under peripneumony becomes greatly increased: if percussion yields a dull sound on the side affected, the probability is again strengthened; and this becomes almost a certainty if respiration ceases to be heard at this part, or if, by means of the cylinder, the *crepitating rale* is heard. Then, in order to complete the diagnosis, the general phenomena of a strong frequent pulse, heat of skin, and thirst, are to be taken into the account.

The first section of this chapter commences with the description of the morbid phenomena of the various systems, in a regular order; the first being entitled *morbid phenomena of the apparatus of digestion, considered as signs.* M. Rostan introduces his details by the following judicious remarks:—

"Let us never forget that an alteration of function announces an alteration in an organ, but let us recollect also that this may be either primitive or consecutive, permanent or temporary. The changes which we are about to mention in the functions of digestion, are at least as often consecutive upon those diseases which have not their situation in those organs, as they are upon a change in the organs themselves. If they are sometimes purely local, they are much more frequently general and sympathetic. But how are these mysterious sympathies brought about? In spite of the efforts of physiologists, nature has not raised the veil which conceals these operations: all we know is, that our organisation, like the machines made by the hand of man, composed of a multitude of parts, is deranged or interrupted if any of these parts becomes displaced, and this in proportion to the importance of the part. Every thing in the human body is tied and linked together, and it is not more surprising to see the circulation disturbed when the stomach is inflamed, than to see the movements of a watch disordered when the regulator is broken." (P. 258.)

"But how are we to distinguish when a phenomenon is merely sympathetic?" Although this be a matter of difficulty, still some general rules may be established. Thus, when it is established in a precise manner that one organ has suffered prior to any other, it will be probable that this is the organ principally affected, and that the functional disturbances which have subsequently arisen are merely sympathetic. Another mode of judging is from the facility with which certain functions are sympathetically affected; and again from the relative position of organs, when the disturbance of the one can be readily explained by the affection of the other. Finally, we are warned not to give implicit confidence to the above methods, but only to look upon them as probabilities, since there is no reason why several affections should not exist at the same time.

M. Rostan finishes this section with a hint to those who attribute all disorders of the digestive functions to a gastro-intestinal irritation; a hint which it is not necessary for us to extract, since it is only valuable to the practitioner on the other side of the Channel.

*On hunger.*—Increase of appetite is very seldom a morbid sign; nevertheless, it is met with in some diseases of the digestive organs, and in some nervous diseases: an example of which is related by our author in the case of a young physician, who experienced sensations of dragging and insupportable weight in the epigastric region, which always gave way, as if by enchantment, when a certain quantity of food was introduced into the stomach. Many other instances have occurred to our author, wherein an excessive degree of hunger was attendant upon organic disease of the digestive organs, but more especially of the stomach itself.

Hunger is commonly a sign of the presence of worms in the intestinal canal: if this is accompanied by twisting or pricking pains in various parts of the abdomen, and if he becomes thinner and paler, this suspicion is increased; but it is only their actual presence in the matters evacuated that can be termed a pathognomic sign of their existence.

In hysteria, an augmentation of appetite is not uncommon. A remarkable instance of this is mentioned:—A girl, named Lhermina, was accustomed to swallow an amazing quantity of food; this hunger was not continual; it returned by intervals. One day, being at the house of a lady, she was suddenly seized with her attack of hunger. In these extremities, she sometimes bit any person who came near her, and her own hands and arms sometimes bore the marks of her bites. On the day above mentioned, she devoured the soup prepared for twenty-four guests who were expected at the house. This girl was hysterical, and had no other symptom of organic disease; she was, however, sometimes attacked with hæmatemesis. Poor girl!

The appetite is also very often greatly increased in cases of mania and hypochondriasis; it is likewise occasionally much augmented before the invasion of acute disease.

Whenever there is a waste to repair, or a more than usual demand for nourishment, as in pregnant women, hunger is not to be considered as morbid. The diminution of appetite is, however, the usual attendant upon most diseases; and since we are sensible that abstinence is one of the most active means of exciting interstitial absorption,—that is to say, of favouring the resolution of all congestions,—ought we to be astonished at the precautions taken to proscribe food in these cases.

Of the deprivation of appetite usually met with in chlorosis, amenorrhœa, &c. a few words are said.

*On thirst.*—As a local sign, thirst is of as little value as want of appetite, but as a sympathetic phenomenon it is of more importance. Its existence is almost always a sign of the presence of irritation, and its intensity has generally a reference to the degree of that irritation. Thus it becomes useful in enabling us to judge of the violence of an inflammation, of the treatment necessary to be employed, and the prognostic to be given. Thirst is usually more intense at the commencement than towards the termination of inflammatory affections; and it sometimes, though rarely, happens that there is no thirst. If in chronic diseases it becomes increased, it is a sign that an irritation has taken place in some organ; but it is to be remembered that it is sometimes provoked by saline substances, by alcohol, by spiced food, and by abundant sweats, &c. Sometimes, where the thirst is considerable, there is still an aversion to liquids. This is the case in hydrophobia. But our author says truly, that this state is not always produced by a contagious virus, for it may arise spontaneously.

*On the teeth and gums.*—A few words will suffice on this head. Grinding the teeth is observed as a sign of many gastric irritations, although it is a healthy and natural action in some few individuals; occasionally it is the precursor of convulsions or delirium. We think our author need scarcely have mentioned the chattering of the teeth in the cold stage of intermittent and other fevers.

With regard to the gums, it is well known that in the scurvy they swell, become red, livid, and bleed; but, M. Rostan says, at an advanced period of life, when the teeth have fallen out, and the gums have become indurated by mastication, they never afterwards assume the spongy appearance, however intense the scurvy may be. In chlorosis, and some other chronic diseases, they are paler than natural, and even diminish in size.

*Of the tongue.*—This organ offers a great number of signs to the physician, but they are of very unequal value, and their importance has been very much exaggerated. The *natural* condition of the tongue is known to every one, but the physician should carefully inquire as to its *habitual* state in the person to whom he is called. "I have known a lady, (observes our author,) whose tongue, in a state of health, is covered with a yellowish white deposit; but, when she is affected with any gastric irritation, it becomes clean." The writer of this article can confirm this remark by quoting his own case, which is very similar. Some individuals, who sleep with the mouth open, have a dry and brown tongue in the morning; others, especially old men, have the root of the tongue of a black colour. Finally, before any opinion is given as to the condition of this organ, it must be remembered that both food and medicine will alter its appearance.

Our author affirms that, although diseases of the digestive organs are painted in a very characteristic manner, the same may equally be said of diseases of the lungs; for in these the tongue changes its colour, and is covered with various coats, as well as in disorders of the alimentary canal.

The signs taken from the state of the tongue being almost always sympathetic, are of course more useful in prognosis than in diagnosis. The tongue is enlarged in several diseases, sometimes so as to threaten suffocation; and, in cases of extreme emaciation, it also becomes diminished in size. In acute diseases, the tongue sometimes appears smaller than natural; it is at the same time dry, rough, red, and pointed, and is occasionally protruded from the mouth with difficulty. This argues a high degree of irritation, either with concentration or diminution of vital power: this is commonly seen in affections of the brain. With regard to dryness of the tongue, the cause of this state is absolutely unknown, but it announces an irritation of the pulmonary or gastric organs. Cracks and fissures in the tongue imply the highest possible state of irritation. A very clear and red tongue announces a violent degree of inflammation. A red, dry, smooth, shining tongue, is sometimes met with at the termination of acute diseases, as well as in those which are more lengthened; and in this latter state they may be looked upon as the sign of a fresh development of inflammatory action.

Upon the whole, we may observe, in conclusion, that there is but little, either of novelty or ingenuity, in what M. Rostan observes of the condition of the tongue, since all its various appearances resolve themselves into signs of irritation. We think that this is the least happy of all the sections we have yet examined; though perhaps this is not so much the author's fault, as it is owing to the little certainty to be generally derived *solely* from the appearance of this organ.

A page or two next ensues, in which the *morbid conditions of the fauces*, and of *deglutition*, are mentioned, but they contain nothing that is not familiarly known; we therefore proceed to consider the act of *vomiting*, as well as the appearance of the matters vomited. These remarks apply equally to nausea, &c. Our author defends M. MAGENDIE's theory of the mode in which vomiting is produced, and continues thus:—

"A still more important pathological conclusion may be drawn from the experiments of M. Magendie; which is, that he succeeded in producing vomiting by introducing into the veins emetic substances. It was not by acting on the stomach, but on the brain, that vomiting was produced, since in these experiments the stomach did not exist, and still that effect was produced. This conclusion is still further proved by the effect of certain odours, or tastes, or even the mere recollection of them, being sufficient to provoke vomiting. Tubercles in the brain give rise to obstinate vomiting; titillation of the uvula produces the same effect. It may therefore be called a cerebral act, caused, indeed, frequently by the state of the stomach." (P. 283.)

Nevertheless, this act may depend upon certain substances taken into the stomach, upon inflammation, cancer, or a certain nervous affection peculiar to it; or, finally, upon the action of some organ which exercises an influence over it.

The consideration of the substances vomited have reference to prognosis, diagnosis, and therapeutic indications; but we do not find any remarks that tempt us to extend our notice of this section, and therefore we pass on to the last process, that of *defæcation;* and the first observation made by our author relates to the necessity of considering those extraneous circumstances which influence the quality of this excretion, especially as to its colour, which is likely to be affected either by certain kinds of food or medicines.

Dejections have been distinguished into critical and symptomatic. The first are followed by a solution or mitigation of many diseases; the latter produces no amendment. It has been the fashion of late to regard all the changes of appearance in the fæces as a sign of intestinal irritation. Our author conceives this to be an error: for example, there is generally costiveness at the commencement of all acute diseases, and this seems to bear some relation to the dryness of the skin under the same circumstance; and yet no one attributes this latter effect to an inflammation of the surface. Neither can we say truly, that the constipation of old men has any relation to inflammatory action. We can readily conceive the mischief to which the long residence of fæcal matter in the rectum may give rise. M. Rostan says, that he has seen the distension equal the size of a child's head. This state is rendered sometimes embarrassing to the physician, because the very inflammation excited by the presence of this quantity of fæces gives rise occasionally to a secretion from the intestinal surface, which, dissolving some of the fæcal matter around it, occasions a kind of diarrhœa; so that the attendants will suppose that the complaint is exactly the contrary to what is really the case. Under such circumstances, M. Rostan has known the intestine to burst, and the contents to be evacuated into the cavity of the abdomen.

Constipation usually takes place when any other excretion is greatly augmented; it is observed in convalescence, and succeeds to the exhibition of purgatives, as a symptom of many other diseases, and sometimes in consequence of the local pressure of a tumor. From the above sketch, it appears that this condition is sometimes sympathetic, but most commonly the sign of a local condition of some of the organs contained in the abdomen; and that, in relation to prognosis and treatment, it is of the highest importance.

With respect to the opposite state, that of increased discharge of fæcal matter, our author denies also that this is the constant effect of intestinal irritation: in fact, he thinks that there are certain conditions of the mucous membrane of the intestines, quite opposed to inflammation, which give rise to very obstinate purgings. The mucous membrane in this state is pellucid, transparent, and as it were œdematous; the mucous matter appears, under these circumstances, to escape mechanically, by the mere feebleness of the mucous glands, or other parts engaged in the performance of this exhalation. Purging sometimes arises towards the conclusion of acute disease; it is a frequent occurrence in dropsies, during dentition, and in many other complaints. In phthisis, it is universally known as a precursor of death: here it arises from ulceration of the mucous membrane.

The gases contained in the intestines merit some attention: they were formerly attributed to the decomposition of the alimentary matters, or were supposed to be taken into the stomach with the food: but these, says M. Rostan, are not the true sources of intestinal gas. In hysteria, the abdomen will sometimes become tumid instantly; this cannot arise from food, because none has been taken in: it must therefore be admitted, that a species of perspiration takes place in the mucous membrane of the intestines, analogous to that which takes place on the skin, and upon the membrane lining the air-passages. In a general way, it may be said that the formation of gas in the intestines is a sign that the gastric organs do not enjoy a perfect degree of energy, though it cannot be considered as a sign of irritation. The uterus is sometimes affected with an accumulation of gas. The very act of passing the stools affords some signs to the physicians, but these are too obvious to require our notice.—On the nature of the fæces themselves, on their colour and consistence especially, M. Rostan makes several remarks; but they are not distinguished by novelty, and are perfectly understood and appreciated by the practitioners in this country.

